# Co-Occurrence of Regulated and Emerging Mycotoxins in Corn Silage: Relationships with Fermentation Quality and Bacterial Communities

**DOI:** 10.3390/toxins13030232

**Published:** 2021-03-23

**Authors:** Antonio Gallo, Francesca Ghilardelli, Alberto Stanislao Atzori, Severino Zara, Barbara Novak, Johannes Faas, Francesco Fancello

**Affiliations:** 1 Department of Animal Science, Food and Nutrition (DIANA), Faculty of Agricultural, Food and Environmental Sciences, Università Cattolica del Sacro Cuore, 29122 Piacenza, Italy; francesca.ghilardelli@unicatt.it; 2 Department of Agriculture Science, University of Sassari, 07100 Sassari, Italy; asatzori@uniss.it (A.S.A.); szara@uniss.it (S.Z.); fancello@uniss.it (F.F.); 3 BIOMIN Research Center, Technopark 1, 3430 Tulln, Austria; barbara.novak@biomin.net (B.N.); johannes.faas@biomin.net (J.F.)

**Keywords:** forage, mold, dairy cow, milk

## Abstract

Sixty-four corn silages were characterized for chemicals, bacterial community, and concentrations of several fungal metabolites. Silages were grouped in five clusters, based on detected mycotoxins, and they were characterized for being contaminated by (1) low levels of *Aspergillus*- and *Penicillium*-mycotoxins; (2) low levels of fumonisins and other *Fusarium*-mycotoxins; (3) high levels of *Aspergillus*-mycotoxins; (4) high levels of non-regulated *Fusarium*-mycotoxins; (5) high levels of fumonisins and their metabolites. Altersetin was detected in clusters 1, 3, and 5. Rugulusovin or brevianamide F were detected in several samples, with the highest concentration in cluster 3. Emodin was detected in more than 50.0% of samples of clusters 1, 3 and 5, respectively. Kojic acid occurred mainly in clusters 1 and 2 at very low concentrations. Regarding *Fusarium* mycotoxins, high occurrences were observed for FB3, FB4, FA1, whereas the average concentrations of FB6 and FA2 were lower than 12.4 µg/kg dry matter. Emerging *Fusarium*-produced mycotoxins, such as siccanol, moniliformin, equisetin, epiequisetin and bikaverin were detected in the majority of analyzed corn silages. Pestalotin, oxaline, phenopirrozin and questiomycin A were detected at high incidences. Concluding, this work highlighted that corn silages could be contaminated by a high number of regulated and emerging mycotoxins.

## 1. Introduction

Mycotoxins are a group of secondary metabolites produced by fungal organisms mostly belonging to the genera *Aspergillus*, *Alternaria*, *Fusarium*, and *Penicillium* strains [1,2,3]. These genera can produce different mycotoxins for which specific regulations have been established in many countries worldwide to protect consumers and livestock from their harmful effects. Approximately 100 countries have specific regulations or detailed guidelines for mycotoxins in food and feed, which are related to aflatoxins (AF) and aflatoxin M1, trichothecenes types A and B (deoxynivalenol or DON, diacetoxyscirpenol, T-2 toxin, and HT-2 toxin), fumonisins, zearalenone (ZEA), ergot alkaloids, ochratoxin A (OTA), patulin, phomopsins, and sterigmatocystin [4]. Different factors play a role in the setting of limits for a mycotoxin in a specific food category, such as availability of toxicological data and occurrence data, socio-economic issues, detailed knowledge about possibilities for adopting adequate sampling and analysis procedures. This information is often present for cereals and other concentrates as well as some of their by-products. Consequently, the regulations or recommendations are mainly issued for these feeds, whereas no legislation is available for any kinds of forages [5,6].

Some recent evidence suggests the greatest ingestion of some regulated mycotoxins by dairy cows could be mainly related to forage contamination [7,8,9], even if this aspect remains insufficiently investigated. In particular, surveys in which researchers investigated the presence of mycotoxins in home-grown forage are very limited when compared to those analyzing the problem of mycotoxin contaminations in cereals [10]. A survey by Driehuis et al. [11] revealed that the contribution of ensiled forage to mycotoxin ingestion in dairy cows on 24 farms in the Netherlands was approximately three times more than that of other feed ingredients. Furthermore, several authors [12,13,14] reviewed different studies from which an estimation of the dietary contribution of whole-plant corn silage to total mycotoxin intake in dairy cows was carried out mainly referring to regulated mycotoxins. However, many other fungal secondary metabolites besides regulated mycotoxins could be detected in forages, even if knowledge on the occurrence of these emerging and not yet regulated mycotoxins in forages appears currently very limited [15,16,17].

Even if most fungi can be eliminated during ensiling, other species such as *Alternaria* spp., *Aspergillus fumigatus*, *Penicillium roqueforti*, *P. paneum*, *F. oxysporum,* and *Monascus ruber* might be able to tolerate high levels of organic acids and carbon dioxide in addition to low availability of oxygen [6,18]. Furthermore, the presence of oxygen in some parts of silage, mainly during the first storage steps or oxygen penetration during feed-out and aerobic spoilage phases, could allow mold growth and mycotoxin production in specific areas of silage [19]. Although lactic acid bacteria are effective in hindering mold growth, just a minor increase in the oxygen concentration could provide the adequate growth conditions for these fungal strains. Indeed, when most of the acetic and lactic acids, as well as carbon dioxide decrease and more oxygen are present, nearly all cereal-associated filamentous fungi may grow [20,21].

As stated by Kung et al. [20], the evaluation of the pH and the quantification of main silage fermentation traits are the basis of evaluating silage fermentation pathways and fermentative quality. For these authors, a further step in the evaluation of silage fermentative quality is measuring other components that are commonly quantified in silages, such as nitrogenous compounds or mycotoxins. As presented previously, this is true for some conventional and regulated in other feeds mycotoxins, such as AF, fumonisins, ZEA, and DON. However, currently available data to connect quality of silage with other kinds of the so-called emerging mycotoxins, characterized by being neither routinely determined nor legislatively regulated, are very scarce [17,22,23,24].

Consequently, the aim of the current survey was to obtain data on occurrence and concentration of different regulated as well as several emerging mycotoxins detected in corn silage to provide information for a comprehensive risk assessment in this largely used forage. The hypothesis to use some microbial groups as biomarkers for characterizing silage mycotoxin contamination was also verified.

## 2. Results

### 2.1. Cluster Analysis Results

The variables used in cluster analysis were total counts of mycotoxins and concentrations of *Aspergillus*-, *Fusarium*-, *Penicillium*-, *Alternaria*-, and other mycotoxigenic fungi produced mycotoxins (Table 1). In particular, the *Fusarium*-produced mycotoxins were grouped as ZEA and its metabolites, trichothecenes type B, fumonisins, and their metabolites (i.e., included hidden fumonisins), enniatins, beauvericin, or other *Fusarium* mycotoxins. No trichothecenes type A were detected in any analyzed samples. Based on the cluster analysis, five different clusters were identified as a result of similarity in quality and quantity of mycotoxins detectable in each sample. An average root mean squared standard deviation of Mahalanobis distance among them of 1.0 was used to identify clusters. The 64 corn silages were, respectively, grouped in cluster 1 (*n* = 24, defined as silages contaminated by low levels of both *Aspergillus*- and *Penicillium*-produced mycotoxins), cluster 2 (*n* = 22, defined as silages contaminated by low levels of fumonisins, and other *Fusarium*-produced mycotoxins), cluster 3 (*n* = 2, defined as silages contaminated by high levels of *Aspergillus*-mycotoxins), cluster 4 (*n* = 9, defined as silages contaminated by high levels of *Fusarium*-produced mycotoxins) or cluster 5 (*n* = 7, defined as silages contaminated by high levels of fumonisins and their metabolites).

### 2.2. Alternaria Produced Mycotoxin in Corn Silages

Counts and concentrations of *Alternaria*-produced mycotoxins were reported in Table 2. Cluster 3 was characterized by the greatest (*p* < 0.05) number of *Alternaria*-produced mycotoxins, whereas the lowest number (i.e., 0.2; *p* < 0.05) was calculated for cluster 2. Altersetin was not quantified in silages belonging to clusters 2 and 4, whereas it was detected in clusters 1, 3, and 5 with incidences on total analyzed samples of 20.8, 50.0, or 28.5%. The average concentrations of altersetin in cluster 1, 3, and 4 were 6.8, 6.9, and 16.4 µg/kg dry matter or DM, respectively. The alternariol and alternariol monomethyl ether were occasionally detected in silages, with the highest incidences reported in cluster 1 (i.e., 21% and 33%, respectively) or cluster 3 (i.e., 100%). Tentoxin was not detected in cluster 3. In other clusters, tentoxin concentrations of 18.2, 46.2, 88.9, and 34.3 µg/kg DM or incidences of 4.2, 9.1, 33.3, or 14.3% were reported for cluster 1, 2, 4, and 5; respectively. Infectopyron was not detected in cluster 4.

### 2.3. Aspergillus Produced Mycotoxin in Corn Silages

Concerning *Aspergillus* produced mycotoxins (Table 2), the number of detected mycotoxins was lower (*p* < 0.05) in clusters 2 and 4 than clusters 3 and 5, the latter being contaminated by more than 4.0 *Aspergillus*-produced mycotoxins. The highest (*p* < 0.05) concentrations of rugulusovin was measured in cluster 3 (i.e., 310.7 µg/kg DM). Either rugulusovin or brevianamide F was detected in the majority of corn silages, with incidence values greater than 88.9%. Similar contamination levels were detected in other clusters. No aflatoxins or nigragillin were detected in any samples. Despite the concentrations of emodin appeared to be very low, being lower than 10 µg/kg DM, it was detected in more than 50.0% of samples for clusters 1, 3, and 5. Kojic acid was not detected in cluster 3, whereas concentrations ranging from 44.1 µg/kg DM to 104.0 µg/kg DM were reported for clusters 1 and 2, respectively. Incidences lower than 21% were calculated for *Aspergillus* produced mycotoxins such as 3-nitropropionic acid, averufin, bis(methylthio)gliotoxin, or asperphenamate. In particular, averufin was detected in one sample of cluster 1 and bis(methylthio)gliotoxin in one sample of cluster 5.

### 2.4. Fusarium Produced Mycotoxin in Corn Silages

Data of different regulated *Fusarium* produced mycotoxins are reported in Table 3.

Concerning fumonisins, clusters 1 and 2 were characterized by lower (*p* < 0.05) fumonisins counts than other clusters (i.e., 4.8 and 5.7 in clusters 1 and 2 with respect to 6.5, 6.7, and 7.7 in clusters 3, 4, and 5; respectively). The regulated FB1 and FB2 were detected in the majority of analyzed corn silages (i.e., occurrence values higher than 95.5%), with the greatest concentrations (*p* < 0.05) measured for cluster 5 (1382.2 and 285.0 µg/kg DM, respectively). Further, high fumonisins occurrences were also observed for non-regulated FB3, FB4, FA1, and masked forms of FA1, their incidences being higher than 54.2% in all clusters. The average concentrations of FB6 and FA2 resulted lower than 12.4 µg/kg DM. The greatest incidences for these two fumonisins were calculated in cluster 5 (i.e., 71.4 and 100.0%, respectively).

Concerning ZEA, this mycotoxin was detected in all clusters, with incidences ranging from 22.2% of cluster 4 to 100.0% of cluster 3. The ZEA concentrations were lower than 42 µg/kg DM without differences among clusters. The zearalenone-sulfone was detected in corn silages belonging to clusters 1, 3, and 5, respectively with concentrations of 91.2, 145.7 and 284.9 µg/kg DM or incidences of 8.3%, 100.0%, and 14.3%.

No trichothecenes type-A were detected in any samples, such as regulated T-2 and HT-2 toxins. Among trichothecenes type-B, DON was detected in 62.5%, 68.2%, 100.0%, 100.0% and 71.4% for cluster 1, 2, 3, 4 and 5; respectively. The greatest concentration was measured in cluster 3 (i.e., 151.7 µg/kg DM). Both nivalenol and deoxynivalenol-3-glucoside were detected at low concentrations and low incidences.

Concerning other than regulated *Fusarium* produced mycotoxins, siccanol, moniliformin, equisetin, epiequisetin, and bikaverin were detected in the majority of analyzed con silages, with incidences higher than 79.2%, except for siccanol in cluster 1 having an incidence of 58.3%. The greatest siccanol concentration (*p* < 0.05) was measured in cluster 4, with a value of 1,269.2 µg/kg DM. Concentrations lower than 20 µg/kg DM were observed for other mycotoxins mentioned above. Chrysogin and rubellin D were not detected in any samples, whereas monocerin was detected in all clusters at low contamination levels. The antibiotic Y was not detected in cluster 4, whereas the 15-hydroxyculmorin was detected in one corn silage belonging to cluster 1. Butenolide and apicidin were detected in clusters 3 and 5. All these aforementioned mycotoxins occurred in concentrations lower than about 20 µg/kg DM.

Enniatins counts were higher (*p* < 0.05) in cluster 3 than others (i.e., 4.5 vs. 1.7, respectively), Concentrations of all enniatins were low (i.e., < 15.2 µg/kg DM). Beauvericin was quantified in the majority of analyzed corn silages (i.e., incidences higher than 83.3%). Lower (*p* < 0.05) beauvericin was measured in clusters 1 and 2 with respect to others.

### 2.5. Penicillium Produced Mycotoxin in Corn Silages

Concerning *Penicillium* produced mycotoxins (Table 4), the clusters 3, 4 and 5 had higher counts than clusters 1 and 2 (i.e., 6.5, 5.4, 6.3 vs. 4.6 and 4.5; respectively).

Ochratoxin A and barceloneic acid were not detected in any samples, whereas 7-hydroxypestalonic, pestalotin, oxaline, phenopirrozin, and questiomycin A were detected at low concentrations (<50.0 µg/kg DM) in several corn silages, with incidences higher than 60%, except for pestalotin that was detected in 45.8% and 27.3% of corn silages belonging to cluster 1 and 2, respectively. The fellutanine A concentrations were higher (*p* < 0.05) in cluster 3 than others (i.e., 577.5 vs. 67.1 µg/kg DM). Mycophenolic acid, as well as its metabolite mycophenolic acid IV were detected only in one corn silage belonging to cluster 1 and one in cluster 4. Other *Penicillium* produced mycotoxins such as skyrin, asperglaucide, chlorocitreorosein, and cyclopenin were sporadically detected in some corn silages belonging to different clusters, even only in very low concentrations (i.e., <10 µg/kg DM).

### 2.6. Other Mycotoxigenic Fungi Strain Produced Mycotoxin in Corn Silages

No mycotoxins produced by fungal strains differing from the previously presented ones were detected in cluster 4, whereas the greatest number (*p* < 0.05) was observed for cluster 3, being 1.50 metabolites from different fungal strains. Ilicicolin E, ilicicolin C, ternatin, fungerin, calphostin, and N-Benzoyl-phenylalanine were not detected in any samples. Even when concentration levels were low (i.e., <10 µg/kg DM), these other mycotoxins were quantified mainly in some corn silages belonging to clusters 1 and 4. Macrosporin was detected in 12.5% of silages grouped in cluster 1.

### 2.7. Corn Silage Characteristics

In Table 5, the chemical, biological, and fermentative parameters characterizing each cluster were reported.

The chemicals did not differ among clusters and appeared in the normal range for corn silage. The starch tended to be greater (i.e., *p =* 0.067) in cluster 3 than others. Concerning fermentative parameters, the pH numerically tended (*p =* 0.156) to be higher in clusters 3, 4, and 5 (i.e., 3.84, 4.02, and 3.83, respectively) than clusters 1 and 2 (i.e., 3.67 and 3.76, respectively). Furthermore, the cluster 4 was characterized by the lowest (*p* = 0.057) acetic acid content (i.e., 2.54% DM), whereas the highest (*p* < 0.05) was measured for cluster 5 (i.e., 3.82% DM). The highest propionic acid concentration was measured for cluster 4, with values of 0.41% DM. Similar concentrations among clusters were reported for concentrations of butyric acid. Numeric differences (*p* = 0.246) were observed for the concentration of lactic acids, being higher than 4.00% DM in clusters 2 or lower than 3.00% DM in cluster 3. Consequently, a lactic to acetic ratio higher than 2 was calculated in cluster 2. The lowest 1, 2 propanediol concentration (*p* < 0.05) was measured in cluster 5 (i.e., 0.17% DM).

### 2.8. Corn Silage Bacterial Communities

Illumina (MiSeq) sequencing of 16S rRNA amplicons generated a data set ranging between 62,451 and 121,586 sequences per sample, after de-noising with dada2 plugin the number of sequences for sample varied between 12,088 and 55,155 raw sequences per sample with a mean length of 417.03 bp. After bioinformatics analysis, a total of 2,005,150 bacterial sequences were classified. Rarefaction analysis showed that the sequencing depth truly reflected the diversity of the microbial communities, as all the samples reached the sequencing plateau (data not shown). A total of 854 Amplicon Sequence Variances (ASVs) were found in corn silage samples after filtering and singletons removing.

**Figure 1:**
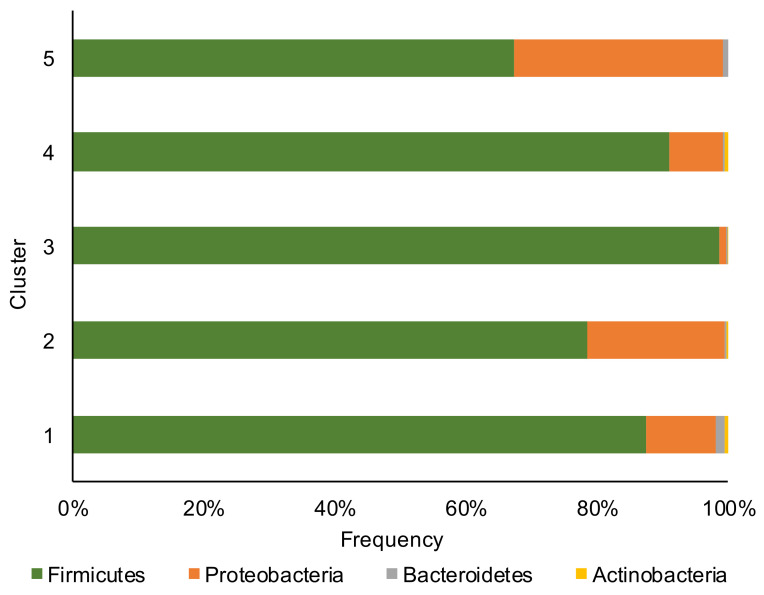
Relative abundance at phylum level of bacterial communities of the corn silage samples grouped in the five different clusters (based on the content and type of mycotoxins).

*Firmicutes*, *Proteobacteria*, *Bacteroidetes,* and *Actinobacteria* phyla (Figure 1) dominated the corn silage samples with the Firmicutes phylum that overcame in abundance all other phyla.

**Figure 2:**
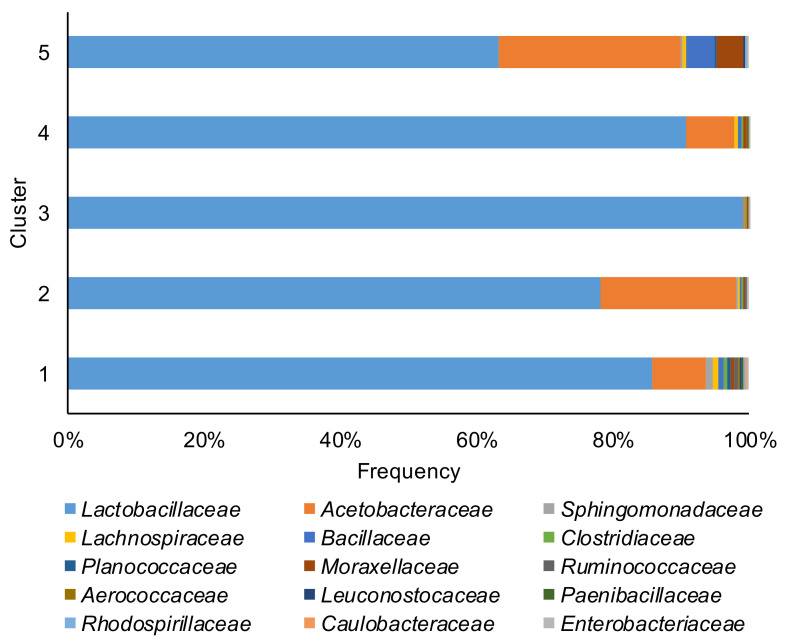
Relative abundance of the top 15 families of bacterial communities of the corn silage samples grouped in the five different clusters (based on the content and type of mycotoxins).

Taxonomic bacterial community profiles at the family level (Figure 2) were dominated by *Lactobacillaceae*, with proportion ranging from 4.3% to 99.9%. *Acetobacteraceae* was the other dominant family with a proportion ranging from 0.3% to 95.6%. In 30 corn silage samples, the *Lactobacillaceae* related ASVs represented 90% or more of all ASVs present. The core microbiome (ASVs present in at least 75% of the samples) of corn silage was composed of four unique ASVs. The species were *Lactobacillus acetotolerans*, *Acetobacter pasteurianus*¸ *Lentilactobacillus buchneri,* and ASVs belong to the *Lachnospiraceae* family. Noteworthy, ASVs affiliated to *Clostridium diolis* that ferment glycerol to 1–3 propanediol [25] were found in 57% of the samples.

**Figure 3:**
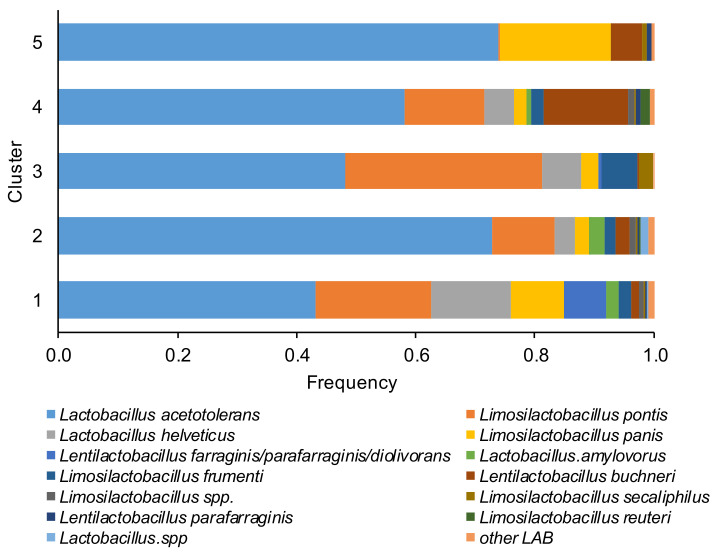
Relative abundance of the lactic acid bacteria (LAB) species of the corn silage samples grouped in the five different clusters (based to the content and type of mycotoxins). The species of LAB were named according to the new classification proposed by Zheng et al. [26].

Based on the last approved lactic acid bacteria taxonomy, the most dominant species in the microbiome of corn silages in a decreased percentage order (Figure 3) were *Lactobacillus acetotolerans*, *Lentilactobacillus buchneri* (formerly *Lactobacillus buchneri*), *Limosilactobacillus pontis* (formerly *Lactobacillus pontis*), *Limosilactobacillus frumenti*, *Lactobacillus helveticus*, *Limosilactobacillus panis*, *Lentilactobacillus farraginis/parafarraginis/diolivorans*, *Lactobacillus.amylovorus*, *Lentilactobacillus parafarraginis*, *Limosilactobacillus secaliphilus Limosilactobacillus reuteri*, and ASVs belong to *Limosilactobacillus* spp. and *Lactobacillus* spp., only a minor number of ASVs were assigned to *Limosilactobacillus coleohominis*, *Limosilactobacillus vaginalis*, *Levilactobacillus brevis*, *Lactobacillus hamsteri*, *Companilactobacillus paralimentarius*, *Lactobacillus delbrueckii subsp. bulgaricus*, *Lacticaseibacillus manihotivorans*, *Liquorilactobacillus vini*.

The percentage of *L. acetotolerans* varied between 43% (in cluster 1) and 74% (in cluster 5), *Limosilactobacillus pontis* between 0% (cluster 5) and 33% (cluster 3), *Lentilactobacillus buchneri* between 0% (cluster 3) and 14% (cluster 4) and *Lactobacillus helveticus* between 0% (cluster 5) and 13% (cluster 1). The cluster 1 was dominated by heterofermentative LAB (*L. pontis*, *L. panis,* and *Lentilactobacillus farraginis/parafarraginis/diolivorans*). Conversely, the homofermentative *L. acetotolerans* dominated the samples of cluster 2. In the clusters 4 and 5 a higher percentage of *L. buchneri* respect to other clusters was detected, while an elevated percentage of the heterofermentative *L. pontis* was found in cluster 3.

**Figure 4:**
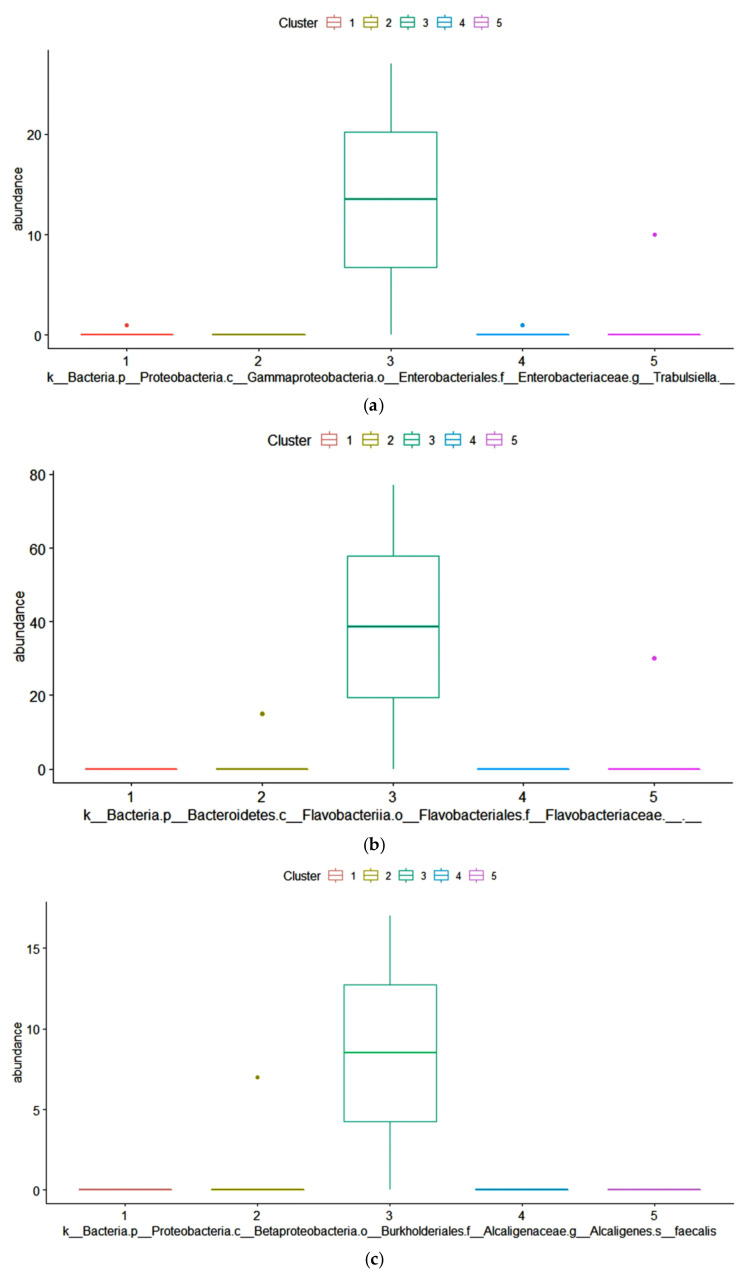
ANOVA analysis with Benjamini–Hochberg False Discovery Rate (FDR) used as multiple test correction method revealed the absolute abundance. The abundances of three Amplicon Sequence Variance (ASVs) (belong to the following taxa: *Trabulsiella*, *Flavobact,* and *Flavobacteriaceae*) were significantly different (*p* < 0.05).

**Figure 5:**
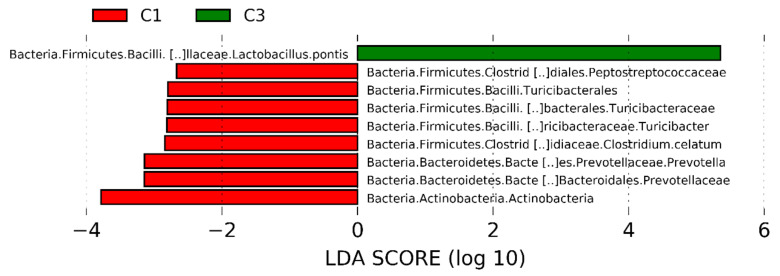
Linear discriminant analysis (LDA) effect size (LEfSe) analysis revealed significant Amplicon Sequence Variances (ASVs) differences in silage microbiota between cluster 1 (red bar) and cluster 3 (green bar). LDA scores (log10) > 2 and *p* < 0.05 are listed.

The dominant ASVs belonging to *Acetobacteraceae* family were affiliated to *Acetobacter pasteurianus* and dominated the microbiome in only 6 samples (>50% of total reads in the sample). In cluster 5, four samples out of 7 had a percentage higher of 25% of *Ac. pasteurianus*. Besides *Lactobacillaceae,* and *Acetobacteraceae*, in 75% of corn silages, the ASVs found were affiliated to *Lachnospiraceae* family, with 57% of samples having an ASV related to *Clostridium dioli*. Cluster 3 was characterized by a great abundance of three ASVs (Figure 4) belonging to family *Flavobacteriaceae*, at genus *Trabulsiella*, and at species *Alcaligenes faecalis* (Figure 4, panel a-c). The Linear discriminant analysis Effect Size (LEfSe) algorithm allowed to identify 9 ASVs as the genomic features characterizing the differences between clusters 1 and 3 (Figure 5 and supplementary figure S1). Particularly *Prevetolla*, *Clostridium,* and *Turicibacter* genus and *Peptostreptococcaceae* family were enriched in samples inside of cluster 1, while the species *Lactobacillus pontis* characterized samples from cluster 3.

## 3. Discussion

### 3.1. Corn Silage Clusterization

To provide comprehensive mycotoxin occurrence data for risk assessment analysis due to corn silage intake, the contamination of several regulated or emerging mycotoxins was determined. Based on mycotoxin screening, the corn silages were grouped in five clusters characterizing by being contaminated by low levels of *Aspergillus*- and *Penicillium*-produced mycotoxins (cluster 1), contaminated by low levels of fumonisins and other *Fusarium*-produced mycotoxins (cluster 2), contaminated by high levels of *Aspergillus*- mycotoxins (cluster 3), contaminated by high levels of no regulated *Fusarium*-produced mycotoxins (cluster 4) or contaminated by fumonisins and their metabolites (cluster 5).

### 3.2. Alternaria and Aspergillus Produced Mycotoxins in Corn Silages

*Alternaria* spp. have been detected in fruits, vegetables, cereals, and derived products whereas few studies focused on silage, as reported in a recent review of its occurrence and role [27]. Storm et al. [28] reported low occurrences and concentrations of alternariol and alternariol monomethyl ether in forages sampled in Denmark, even if occurrence data were reported for other *Alternaria* produced secondary metabolites. Some *Alternaria* species were detected in hay and silage, as *A. alternata*, *A. arborescens* and *A. tenuissima* [18,29]. *A. alternata* was also considered as rare [30], most strains originally identified and attributed to *A. alternata* belonging to *A. tenuissima*, *A. arborescens* or other *Alternaria* species-groups. Alternariol, altertoxins, altenuene, tentoxin, and tenuazonic acid, and a broad range of compounds are produced by these species even with unconfirmed toxic properties. Escriva et al. [27] recently focused on altertoxins and macrosporin. However, *Alternaria* spp. can produce more than 70 different toxins including other secondary metabolites, such as 4Z-infectopyrone, phomapyrones, novae-zelandins, dehydrocurvularin, pyrenochaetic acid or alternarienonic acid produced by *A. infectoria* [30,31]. The main *Alternaria* produced mycotoxins detected were altersetin, alternariol, alternariol monomethyl ether and 4Z-Infectopyron, even only at low contamination level (<30 µg/kg DM) and low incidence. However, tentoxin contaminated 3 corn silages grouped in cluster 4 at an average concentration of 88.9 µg/kg DM. Streit et al. [23] reported also the occurrence of tentoxin, altenuene, and tenuazonic acid in feed and silages, thus confirming present findings. *Alternaria* produced toxins have been shown to have harmful effects in animals, including cytotoxicity, fetotoxicity, and teratogenicity, and they can cause also from hematological disorders to cancer due to their mutagenic nature [6,27]. However, toxicological data on *Alternaria* produced mycotoxins are limited.

The most important mycotoxins produced by *Aspergillus flavus* and *A. parasiticus* were AF and they were identified occasionally and at low levels in silage. No aflatoxins were detected in any corn silage samples, which is in line with previous findings [11,12,32]. Kojic and 3-nitropropionic acid are also produced by *A. flavus* and other *Aspergillus* species [33]. Their incidences were low in all samples, except in corn silages belonging to cluster 5. Their concentration could be considered low, being equal to 105 µg/kg DM for kojic acid and 6.8 µg/kg DM for 3-nitropropionic acid. In the Netherlands, Santos and Fink-Gremmels [34] detected in one of three sampled silages a concentration of 3-nitropropionic acid much higher of 1360 µg/kg.

Silages are often contaminated by *Aspergillus fumigatus* related toxins [18,35,36]. Among 226 potentially bioactive secondary metabolites produced by this mycotoxigenic fungus, gliotoxin is the most toxic one and it often reported to contaminate corn silages [37,38]. No gliotoxin was detected in any samples, whereas its metabolite bis(methylthio)gliotoxin was found only in one sample of cluster 5, which corresponds to findings from our previous survey [39,40]. Either rugulusovin or brevianamide F were detected in the majority of analyzed samples and could be produced by different mycotoxigenic filamentous fungi belonging to *Aspergillus* spp. and other strains. In particular, rugulusovin is a secondary metabolite of *A. halophilicus* [41] and brevianamide F could be produced by *A. clavatus* [42]. Two corn silages belonging to cluster 3 were highly contaminated by these mycotoxins, resulting in average concentrations of 310.7 and 246.4 µg/kg DM. As their toxic effect is mostly unknown in livestock, and their presence in corn silages is notable, future studies are needed to clarify the actual risk of their ingestion by farm animals. Other *Aspergillus*-produced mycotoxins detected in corn silages (i.e., emodin, averufin, and asperphenamate) contaminated only irregularly the analyzed samples and at very low concentrations.

### 3.3. Fusarium Produced Mycotoxins in Corn Silages

*Fusarium* mycotoxins are primarily produced by *F. proliferatum* and *F. verticillioides* and the FB1 and FB2 appeared to be the most predominant among fumonisins [43]. Their incidences were reported to be higher than 30% in corn silages sampled in North America [29,44,45], whereas lower incidences were reported in the Netherlands and France [12,46]. In this current survey, FB1 and FB2 were detected in almost all collected silages. High contamination levels of FB3 were also detected in corn silages [45,47]. Concerning other fumonisin metabolites, the FB4, FB6, FBA1, and FBA2, as well as masked fumonisin forms, the data regarding their incidences in corn silages are still scarce. As fumonisin contamination in corn silage is usually related to pre-harvest crop conditions [11,18], the specific environmental conditions characterizing the sampling years, as well as the crop management strategies before harvest, can have promoted fumonisin contaminations, with the exception for FB6 and FBA2 that showed low incidences data. Despite this, the level of fumonisin contamination appeared to be low and safe for lactating cows [9], except in cluster 5.

Different authors did not detect ZEA in corn silages [38,40], on the contrary, previous surveys often identified ZEA in corn silages, although at a concentration lower than 500 µg/kg [12,48,49,50]. These results seem to confirm present findings, in which the ZEA concentration was lower than 300 µg/kg DM for all clusters.

Concerning trichothecenes type B, they are primarily produced by *F. culmorum* and *F. graminearum* before harvest [43] and are usually detected in corn silages. In particular, DON is the main mycotoxin in silages and other forages within a broad range of concentration and occurrence [18,51], whereas nivalenol and fusarenon-X, as well as their acetylated and deacetylated analogues (3- acetyl-DON, 15-acetyl-DON and others) were detected at a less extent. Varying contamination levels of DON in forages were reported in North America and North Europe [12,28,49], sometimes exceeding 2000 µg/kg and with incidences higher than 80% [47]. On the contrary, the occurrence of nivalenol was much lower in corn silage [28,49]. These data appeared in line with those reported in a current survey, in which DON was detected in the majority of analyzed samples even at low concentrations (i.e., at most 151.7 µg/kg DM in cluster 3), whereas nivalenol occasionally contaminated corn silages independently by clusters.

Type A trichothecenes such as diacetoxyscirpenol (DAS), T2, HT2 toxins and their de-acetylated analogues from *F. poae*, *F. sporotrichioides,* and *F. langsethiae* [43] were often detected in corn silages [28,49,50] but were not detected in analyzed samples from this survey, thus showing the risk related to their ingestion is very low in specific sampling areas.

Among other *Fusarium*-produced mycotoxins, a high incidence of siccanol, moniliformin, equisetin, epiequisetin, and fusaric acid was observed. The corn silages belonging to cluster 4 were contaminated by these kinds of mycotoxins at high level, in particular regarding siccanol and fusaric acid. Streit et al. [23] reported that 29% of feed samples were contaminated with siccanol with median and maximum values of 9607 and 39,850 μg/kg, respectively. Similarly, fusaric acid was detected in 26% of feed samples with median and maximum values of 643 and 13,593 μg/kg. The fusaric acid (mean = 765 µg/kg) was detected in high prevalence and concentration in Israeli corn silage and it was defined as the toxin with the highest potential concern to dairy cow performance [52]. More recently, Ekwomadu et al. [53] found that siccanol occurred in 74% of 120 maize samples with a mean value of 64 μg/kg (i.e., range from 35 to 252 μg/kg). In the same survey, fusaric acid was found in 20% of 120 samples with a mean value of 85 μg/kg (i.e., range from 58 to 195 μg/kg). As stated by these authors, the co-occurrence of these emerging mycotoxins with other major mycotoxins and many other *Fusarium* metabolites of unknown toxicity is still a source of concern and should be studied in more detail. In particular, the ability of the rumen to denature these toxins might be impaired by the high passage rate in high-yielding dairy cows and mixture of toxins could end in a synergistic interaction. The presence of DON and fusaric acid in vitro caused depression of both *Ruminococcus albus* and *Methanobrevibacter ruminantium* microbial activity, whereas these effects were not observed in the presence of DON alone [54]. Custódio et al. [55] found levels of fusaric acid in corn silage used in beef cattle diets of 619 µg/kg with high incidence (i.e., 80%). The same authors highlighted that fusaric acid increased toxicity of other *Fusarium*-produced toxins through a synergistic mechanism [1] and this required further studies to be completely elucidated.

Similar to results reported in current survey, beauvericin and enniatins were detected at very low contamination levels in silages from Ireland and Denmark [28,56,57]. Ekwomadu et al. [53] also found an occurrence of beauvericin in 87% of 120 samples of maize grains (7.2 and 142 µg/kg for mean and max levels), whereas enniatins were detected only in less than 2% of samples.

### 3.4. Penicillium Produced Mycotoxins in Corn Silages

Predominant presence of post-harvest fungi in silages is associated to the presence of *P. roqueforti* and *P. paneum* [46,58,59,60]. These species belong to *Penicillium* section *Roqueforti* [61] and are able to tolerate typical silage conditions, such as high CO_2_, low pH, and oxygen. In particular, unfavorable weather or storage conditions during plant growth or at different ensiling phases could stimulate fungal proliferation and mycotoxin production in silage [46,62]. Nielsen et al. [63] listed the main mycotoxins produced from *Penicillium* strains, which are also more intensely studied and highly detected in forages. In particular, *P. roqueforti* was isolated from 89% of visibly moldy and from 85% of visibly un-moldy silages [64]. In the Netherlands, Driehuis et al. [12] reported an incidence of 50% for mycotoxins produced by *P. roqueforti* in corn silages. Among mycotoxins produced from *Penicillium* in silage, mycophenolic acid and roquefortines are most frequently detected [7,64,65]. On the contrary, Storm et al. [28,60] reported less than 3% of collected samples resulted being contaminated by these *P. roqueforti* produced mycotoxins, and these findings resulted in line with those reported in the current survey. In particular, mycophenolic acid and mycophenolic acid IV were detected in some samples of clusters 1 and 5. The concentrations appeared to be low and far to those levels of contamination associated to animal disorders [6]. Other exometabolites of *Penicillium* spp., such as the *P. paneum* biomarker marcfortine A, festuclavine, agroclavine, andrastin A, and citreoisocumarin, were also found in silages [7,28]. Some toxins related to secondary metabolism of *Penicillium* strains were detected in the analyzed corn silages, being secalonic acid, 7-hydroxypestalonic, pestalotin, oxaline, fellutanine A, phenopyrrozin, or questiomycin A. The concentration of these *Penicillium* produced mycotoxins appeared negligible, except for fellutanine A, a bio-active diketopiperazine alkaloid isolated from the cultures of *Penicillium fellutanum* that belongs to a class of naturally occurring 2,5-diketopiperazines. The fellutanines A−C, are non-annulated analogues of cyclo(L-Trp-L-Trp), but unlike their diannulated analogue fellutanine D are not cytotoxic. Consequently, further research is required to verify the real effects of these *Penicillium* related mycotoxins on animal health and on production performance.

### 3.5. Other Fungi Strain Produced Metabolites in Corn Silages

Illicolins are fungal metabolites produced by different strains of *Fusarium* spp. and other pathogenic fungi of different cereal crops, such as *Ascochyta* and *Cylindrocladium* spp. Illicolins led to cytotoxic effects on HeLa cells, but exerted only limited antibacterial activity. Subsequent studies showed that they were also toxic to various yeasts, thus showing an antibiotic effect [66]. Illicolin A and B were sporadically detected in corn silages. Ascochlorin is an isoprenoid antibiotic that is produced by the phytopathogenic fungus *Ascochyta viciae* [67]. Macrosporin is a bioactive metabolite produced by *Stemphylium lycopersici* causing leaf necrosis in plants [68]. These compounds were detected in corn silages as secondary metabolites of different fungal strains, but their effects on animal health are still unknown.

### 3.6. Corn Silages Chemical Characterization

The chemical composition of corn silages belonging to different clusters did not differ and the average values, as well as ranges of measurements could be considered in line with our previous survey [39,69] and with those reported in other nutritional tables. The fermentative traits slightly differed among clusters showing different fermentation quality of corn silages. In particular, average values of pH higher than 4.0 were measured for cluster 4 and this limit appears critical to identify poorly made corn silage contaminated by pathogenic bacteria [70]. For this cluster, the numeric (*p* > 0.158) high presence of butyric acid could be related to the metabolic activity of both saccharolytic and proteolytic *Clostridium* spp. [71,72]. The other clusters characterized by having a high level of mycotoxin contamination were clusters 3 and 5. Even if the pH resulted below the 4.0 threshold value, it appeared to be higher than that measured for clusters 1 and 2. Probably due to higher DM contents characterizing the two silages belonging to cluster 3, the concentrations of lactic acid were lower than for other clusters, thus reducing the lactic to acetic ratio to values below 1.0, usually defined as a threshold value for characterizing abnormal fermentation in silages [20]. For cluster 5, the high pH and the low lactic to acetic ratio as well as ammonia-nitrogen concentrations could be associated with a poor fermentation quality of these corn silages. In particular, Huhtanen et al. [73] reported the negative correlation between propionic acid and ammonia-nitrogen with digestible organic matter in lactating dairy cows. These altered traits could be probably associated with poor fermentation that produce these end-products during fermentation. Furthermore, the presence of *F. verticillioides* was associated with production of aldehydes and other volatile compounds [56].

The corn silages belonging to clusters 1 and 2 were less contaminated by different types of mycotoxins and their fermentation profile, characterized by low pH, low clostridia associated end-products, and high concentrations of both lactic and acetic acids, could be considered adequate [20]. These interpretations of fermentative profile appeared in line with the higher use of bacterial inoculants registered for corn silages belonging to these clusters, as well as the use of commercial products containing *L. buchneri* most common in cluster 1 than cluster 2. The level of ethanol measured in these corn silages did not differ with other poor preserved samples, thus confirming previous findings reported for corn silages and for which bacterial inoculants did not reduce ethanol [74,75,76,77].

### 3.7. Corn Silage Bacterial Communities

#### 3.7.1. *Lactobacillaceae* Community in Corn Silages

Several authors showed that the dominant bacteria at the end of the fermentation phase of different types of silages belong to the family of *Lactobacillaceae* [78,79,80,81,82]. In general, in properly ensiled forage, facultative anaerobic bacteria gradually decrease silage pH, promoting the growth of acid-tolerant LAB, which typically dominate the eubacterial microbiome of terminal silage [72,78]. Corn silage samples analyzed in this work showed a low microbial biodiversity, and several samples were dominated by unique ASVs, and no significant differences were noticed between the five clusters in regards to the *Lactobacillaceae* community. This was more pronounced at the feed-out phase, as the aerobic exposure causes a decrease in microbial biodiversity [78,83]. Duniere et al. [78] observed an increase in *Lactobacillaceae* Operational Taxonomic Unit (out) in barley silage and hypothesized that some of the species were in a viable, but nonculturable state, with these populations increasing during aerobic exposure. Taxonomic identification of key ASVs sequences was performed by comparing the sequences to the National Center for Biotechnology Information (NCBI) database. After a blast search of dominant ASV (935,209 reads), the highest scoring hits against the sequence of the ASV (427 bp, % identity 100%) affiliated to *Lactobacillus acetotolerans*. This species is a facultative heterofermentative lactic acid bacteria (some strains utilize ribose), that presents a high resistance to acetic acid [26]. Xu et al. [82], sequencing the full-length 16S rRNA gene of whole crop corn silage bacteria using PacBio SMRT technology, found *Lb. acetotolerans* as the dominant species after 90 days of fermentation. Interestingly, the same authors also found a positive correlation between *Lb. acetotolerans* with 4-hydroxybutyrate, azelaic acid and 3-phenyllactic acid contents. All these compounds showed antimicrobial activity. In particular, the latter has a strong antifungal activity [84]. However, further studies will be needed to fully elucidate *Lb. acetotolerans* related to its antifungal activity in silages.

The latest approved taxonomy was used for the classification at species level of lactic acid bacteria [26]. The most dominant species in the microbiome of corn silage were *L. acetotolerans*, *L. helveticus* and *L. buchneri*. Indeed, the latter is one of the most regularly used LAB in silage inoculants due to its capacity to synthesize acetic acid even under low pH [83]. Acetic acid as a strong antifungal activity increases the aerobic stability of silage. Noteworthy, according to Drouin et al. [81], we found the *Lb. helveticus* as part of the common lactic bacteria community. As components of lactic bacteria community, we also found *Lim. secaliphilus*, *Lim. panis*, and *Lim. pontis,* which were isolated from type I and II sourdoughs, and the last species also from intestinal microbiota of swine, silage, dairy products, mezcal fermentation, and wet wheat distillers’ grains [85,86]. The habitat of *Lim. secaliphilus* remains unknown [87]. These species, which belong to the group of *Lim. reuteri*, might play a role in the metabolism of 1–2-propanediol (Sriramulu et al., 2008). *Lim. panis* can degrade lactic acid and acetic acid. However, Li and Nishino [88] found that *Lim. panis* was present in the low lactic acid samples. Other minor species found in corn silage were *Len. farraginis* and *Len. parafarraginis*, belonging to *Len. buchneri* group. The last species was already used as a silage inoculant and was found to be able to increase acetic acid content and improving the aerobic stability of silages in a wide range of temperature [89,90,91].

#### 3.7.2. *Acetobacteriaceae* Community in Corn Silages

Concerning other bacterial communities, the presence of *Acetobacteraceae* is considered undesirable because it can initiate aerobic deterioration and degrade lactic acid and acetic acid to produce carbon dioxide and water [92], even if contrasting results were presented in the literature. *A. pasteurianus* does not produce acetic acid under anoxic conditions [93] and only in the presence of residual oxygen and ethanol, it can convert ethanol to acetic acid [94,95]. *A. pasteurianus,* together with species such as *Klebsiella variicola, Enterobacter hormaechei*, and *Bacillus gibsonii,* were found in deteriorated Italian ryegrass, guinea grass, and whole-crop maize silages (Li and Nishino, 2013). Li et al. (2011) found that *A. pasteurianus* was uniformly distributed in the three maize silage stored in bunker silos. The same authors asserted that none of the farmers acknowledged problems with these silages. Li et al. [96] suggested that acetic acid bacteria could be used to improve silage aerobic stability. Previous authors [95,96] reported that *A. pasteurianus* treatment did not affect the aerobic stability of corn silage. Given the ability to fixing nitrogen of this bacteria [93], it would be worth evaluating their role during the feed out on the silage microbiome.

#### 3.7.3. Other Bacterial Family Community in Corn Silages

The differential family ASVs abundance analysis among the five clusters showed that in cluster 3, the cluster with the higher content of *Aspergillus*-produced mycotoxins, had a higher abundance of three ASVs belonging to the *Flavobacteriaceae* family (Figure 3), to the genus *Trabulsiella* (Figure 4) and to the species *Alcaligenes faecalis* (Figure 5) respectively. In particular, the family of *Flavobacteriaceae* is the largest family in the phylum *Bacteroidetes* [97] in which most are aerobic bacteria. Overall, in our samples, the ASVs abundance of this family is below 1%. *Flavobacterium*, a genus belonging to *Flavobacteriaceae* family, was found in silage because of its ability to produce cellulase [98] and release soluble carbohydrates, substrates available for other microorganisms. *Alcaligenes faecalis* is a gram-negative species that degrades urea and releases ammonia, thus increasing pH. This might decrease the aerobic stability of the silage and favor the fungi development. Dutkiewicz et al. [99] found that *Alcaligenes faecalis* is the most common species in bulk silage. Intriguing, Zhang et al. [100] reported that this bacterium has the ability to degrade ochratoxin A through the activity of N-acyl-L-amino acid amidohydrolase enzyme. *Alcaligenes faecalis* N1-4 isolated from tea rhizosphere soil is able to produce abundant antifungal volatiles, such as dimethyl disulfide (DMDS) and methyl isovalerate, and greatly inhibited the growth of *A. flavus* [101]. Therefore, the higher concentration of mycotoxin in cluster 3 samples might have stimulated the growth of these bacteria.

*Trabulsiella*, a genus of the family *Enterobacteriaceae* [102], has been found in different habitats. *Trabulsiella* strains have the genetic machinery for heterotrophic, aerobic metabolism and, like most close relatives, all genes for glycolysis, the tricarboxylic acid (TCA) cycle, the pentose phosphate pathway, and oxidative phosphorylation. Like their close relatives, *Trabulsiella* strains have the machinery for dissimilatory nitrate reduction (nitrate to nitrite to ammonia). Sapountzis et al. [103] found that *T. odontotermitis*, a facultative symbiont of termite’s gut, might play a role in carbohydrate metabolism and aflatoxin degradation. Suman et al. [104] found the strain of IIPTG13 *Trabulsiella* also might play a role in the lignin degradation. Recently, Olvera-García et al. [105] described the genome of five strains of *Trabulsiella* and found that this species has a pool of genes related to cellulose degradation. Maybe at the feed-out an aerobic microbial community capable of degrading cellulose settles down after the levels of soluble carbohydrates decrease.

The LEfSe analyzes allowed to detect several ASVs as biomarkers of samples of cluster 1, in particular, several strictly anaerobic species were enriched in samples of cluster 1 with respect to clusters 3, 4, and 5. The decreasing number of strictly anaerobe species in samples silage of others clusters might be due to the higher aerobic exposition of these samples, which leads to their decrease or disappearance. Therefore, the strictly anaerobic species could be used as biomarkers relate to the aerobic exposure time of silage.

### 3.8. Priorities for Future Investigations

Summarizing the contribution knowledge advance from this survey, a description of the presence and concentrations of emerging mycotoxins in silages has been presented. The limits of this work are mainly related to its descriptive structure not specifically linked to a focused hypothesis on the growth and occurrence of emerging mycotoxins. On the other hand, considering the little information reported in literature about these compounds, the correlation of their presence and incidence with silage characteristics and microbial communities, can be considered as a relevant contribution to the field of silage evaluation and mycotoxin risk analysis for dairy cows. More information is strongly requested to better describe the possibility to prevent the occurrence of these compounds and also to define their biological role, since the knowledge is very limited [17].

From an applicative point of view related to ruminant farms and nutritional effects, priorities of future investigation should be oriented toward two main areas: understanding of the field and silage management conditions favoring the development of emerging mycotoxins, their relations with biomass composition and environmental conditions, both at an agro-technical level and during the ensiling processes in the farm bunk. In this sense, as provided for regulated compounds [6], it is essential to deepen knowledge on the physicochemical environment of ensiling conditions favoring fungal development and mycotoxins production and moreover, on practices that could help to prevent silage contamination. Specific focus should be on the effects of pH and volatile fatty acid (VFA) concentrations in the modulation of fungal growth. As reported for regulated compounds [19], further investigations are also needed on the specific areas of the bunk that could be more affected by emerging mycotoxins.

More research is needed for a better understanding of the effects on the animal performance (intake and feed behavior, production, reproduction, health) of different ingestion doses of emerging mycotoxins, or on interactions and synergistic effects of emerging and regulated compounds that could be detrimental for animal production and human health [1].

## 4. Conclusions

The manuscript presents comprehensive mycotoxin occurrence data useful for risk assessment analysis due to the ingestion of contaminated corn silage. Based on mycotoxins detected in silage, different groups of contamination were defined and they were silage contaminated by low levels of *Aspergillus*-, *Penicillium*-produced mycotoxins; by low levels of fumonisins and other *Fusarium*-produced mycotoxins; by high levels of *Aspergillus*- mycotoxins; by no regulated *Fusarium*-produced mycotoxins or contaminated by fumonisins. More than chemicals, some fermentative traits, and different bacterial communities could be related to a different quality of these silages, thus supporting the idea that the safety of such type of forage and the concentration of these regulated and emerging mycotoxins can be modulated by different dynamics of forage colonization of specific bacterial groups. In particular, the dominant bacteria detected in all silages belong to the family of *Lactobacillaceae*, with the most abundant one being *Lactobacillus acetolerans*, followed by *Acetobacteraceae*. Furthermore, we observed that different not dominant bacterial communities are related to the quantity and quality of regulated and emerging mycotoxins contaminated silages. In particular, the production of organic acids with antifungal properties could improve the aerobic stability of the silages representing a positive aspect. Otherwise, some microbial groups can use organic acids as substrates for their metabolisms, favoring the aerobic deterioration of silages and mold proliferation. As a result of this survey, the use of some microbial groups as biomarkers for characterizing silage quality and mycotoxin contamination, such as strictly anaerobic bacteria, was supported even if further investigations are needed. Other important aspects are the role of good practices in silage cultivation, harvesting, and conservation to reduce the risk of fungal growth and (emerging) mycotoxin production. Finally, information is still lacking regarding the role of emerging mycotoxins, alone or in combination with regulated ones, on animal performance and their health status, which represents a novel and unexplored area of research.

## 5. Materials and Methods

### 5.1. Sample Collection, Preparation and Analysis

Sixty-four dairy farms located in the Po Valley (Italy) and Sardinia were randomly selected and visited in the 2017–2019 harvest seasons to collect corn silage samples. Corn silages were sampled at least 10 weeks after ensiling from horizontal bunker silos in agreement with the sampling procedure already described by Gallo et al. [40]. Samples of about 1.5–2 kg on a wet weight basis were sampled from at least 4 points of each silage feed-out face and analyzed as describe below.

All collected samples were split into 2 sub-samples of about 750–1000 g/sample of fresh matter. A sub-sample was treated as previously described by Gallo et al. [69] for chemical, biological, and mycotoxin analysis or by Gallo et al. [40] for characterizing fermentative traits. Briefly, samples were dried at 60 °C in a ventilated oven for 48 h until constant weight, then milled through a 1-mm screen using a laboratory mill (Thomas-Wiley, Arthur H. Thomas Co., Philadelphia, PA, USA) and stored until analysis.

All corn silages were analyzed for the presence and concentrations of fungal metabolites by LC–MS/MS at the Department of Agrobiotechnology according to Sulyok et al. [106]. As recently described by these authors, the analytical method has been extended to cover more than 500 metabolites. Briefly, 5 g of sample was weighed and extracted with 20 mL acetonitrile/water/acetic acid (79:20:1, v/v/v) for 90 min on a rotary shaker (GFL, Burgwedel, Germany). Extracts were diluted in extraction solvent (ratio 1:1) and directly injected into the LC–MS/MS instrument. All chromatographic characteristics were previously described [106]. Mycotoxins were quantified by external calibration (1/x weighted) using a multi-component standard prepared from authentic standards.

The DM was determined by gravimetric loss of free water by heating at 105 °C for 3 h (Association of Official Analytical Chemists or AOAC 1995, method 945.15); ash was determined as gravimetric residue after incineration at 550 °C for 2 h (AOAC 1995, method 942.05) and ether extract (EE) was obtained following the method 920.29 of AOAC (1995). The crude protein (CP, N × 6.25) was determined using the Kjeldahl method (AOAC 1995, method 984.13). The soluble fraction of CP (expressed on a DM basis) was determined according to Licitra et al. [107]. The neutral detergent fiber (NDF), acid detergent fiber (ADF), and lignin (ADL) were determined using the AnkomII Fiber Analyzer (Ankom Technology Corporation, Fairport, NY, USA) according to the method described by Van Soest et al. [108]. The NDF analyzes utilized a neutral detergent solution containing sodium sulfite and a heat-stable amylase (activity of 17.400 Liquefon units/ml, Ankom Technology). The NDF, ADF and ADL contents were corrected for the residual ash content. Starch was measured by polarimetry (Polax 2L, Atago^®^, Tokyo, Japan). The disappearance of NDF after 24 h of rumen incubation (24 h NDFD) was measured in situ by incubating nylon bags in the rumen of two cannulated dry cows for 24 h [109]. Cows were daily fed about 10 kg DM of a total mixed ration consisting of alfalfa hay, ryegrass hay, corn silage and concentrate (i.e., 250, 350, 300, and 100 g/kg DM, respectively) in two portions at 8:00 a.m. and 6:00 p.m. The diet contained 120 g CP/kg DM and 550 g NDF/kg DM.

Immediately after collection, the second sub-sample was stored as wet at 4 °C and analyzed within 24 h for acetic acid, propionic acid, butyric acid, lactic acid, NH3-N, and pH. In particular, about 50 g of wet samples were extracted using a Stomacher blender (Seward Ltd., West Sussex, UK) for 3 min in distilled water at a water-to-sample (fresh weight) ratio of 3:1. The water mixture was then filtered through a gauze and an aliquot (10 mL approximately) of the resulting solution was centrifuged at 4500× *g* for 15 min. The liquid supernatant was micro-filtered on a 0.45 µm syringe filter and an aliquot of micro-filtered supernatant (1.5 mL) was transferred in a vial and added with 10 µL of internal standard solution (pivalic acid at 1.5% in distilled water) for VFA gas-chromatographic analysis [76]. The analysis was carried out using a 2025 GC with Flame Ionization Detector (FID) system (Shimadzu S.r.l., Milano, Italy) equipped with auto-sampler (AOC-20i Shimadzu S.r.l., Milano, Italy) and capillary column DB-FFAP (30 m 0.250 mm; 0.25 µm; Agilent Technologies S.p.A., Milano, Italy). The analysis conditions were as follows: 200 and 230 °C injector and detector temperatures, respectively; 1.5 mL/min constant flow of hydrogen as carrier gas; 1 µL injection volume; and 30:1 split ratio. The temperature program was 40 °C 5 min, after 60 °C at 10 °C/min 5 min, after 160 °C at 5 °C/min, after 200 °C at 3 °C min. The VFA in silage samples were identified with the aid of external standards and calculated through peak areas corrected by factors of response instrumental and using pivalic acid as an internal standard. The micro-filtered extract used for volatile organic compounds (VOC) analysis was diluted (1 + 9) with 8.0 mM H2SO4 in a vial and injected into HPLC for lactic acid determination. The HPLC system consisting of PU-2080 pump, an AS-2055 sampling system, and a UV-2070 detector. An organic acid analysis column (Amine^®^ HPX-87H Ion exchange column, 300 mm × 7.8 mm i.d., Bio-Rad, Hercules, CA, USA) was used at 35 °C with a mobile phase of 8.0 mM H2SO4 at 0.6 ml/min. The UV detector was set at 210 nm. The quantification of lactic acid was done using an external standard.

The NH3-N was determined from about 20 g of fresh samples in a slurry mix composed of 150 ml of distilled water and magnesium oxide (10 g/sample). The NH3-N content, expressed on a total nitrogen (TN) basis, was determined after steam distillation of this solution by the Kjeldahl method as reported above. Finally, the pH value was measured on the previously filtered water mixture.

### 5.2. Bacterial Genomic DNA Extraction and Bioinformatics

About 50 g of wet samples were homogenized as described above, using a Stomacher blender (Seward Ltd., West Sussex, UK) for 3 min in distilled water at a water-to-sample (fresh weight) ratio of 3:1. After homogenization, the sample was centrifuged for 15 min at 4600× *g*. The supernatant was discarded and the pellets were resuspended in 3 mL of double distilled sterile water. Aliquots of 1.5 mL of this suspension were centrifuged at 21,000× *g* for 3 min and the obtained pellets were used to extract the bacterial genomic DNA by the DNeasy^®^ PowerSoil^®^ Kit (QIAGEN, Santa Clarita, CA, USA), according to the manufacturer’s instructions. The extracted DNAs were stored at −80 °C until further analysis. The variable region V3-V4 of the 16S rRNA gene was amplified from DNA samples by PCR using the primer pair 341F (CCTACGGGNGGCWGCAG) and 805R (GACTACHVGGGTATCTAATCC) and sequenced by the 400 Illumina MiSeq platform (Paired-end, 2 × 301 bp, Macrogen, Seoul, Korea).

The Paired-End Illumina MiSeq reads (demultiplexed FASTQ files) were analyzed with the Quantitative Insights into Microbial Ecology tool (QIIME 2 version 2019.10). Sequence quality filtering and Operational Taxonomic Unit (OTU) picking were carried out using the Dada2 plugin wrapped in QIIME2 [110]. Taxonomic classification of 16S Amplicon Sequence Variant (ASV) obtained by denoising analysis was carried out using a pre-trained Naive Bayes classifier trained on the Greengenes 13_8_99% OTUs. The phylogenetic tree for diversity analysis was constructed using the q2-fragment-insertion plugin. To account for uneven sequencing depth between samples the data were normalized (the rarefaction depth was set at 12088 sequences/sample) using the diversity QIIME alpha-rarefaction plugin. Diversity analysis was carried out using the QIIME diversity core-metrics-phylogenetic plugin. To investigate the microbial diversity differences between clusters the QIIME diversity alpha-group-significance and beta-group-significance plugin were used. Data were visualized using the QIIME dedicated plugin. Linear discriminant analysis Effect Size (LEfSe) algorithm [111] was applied to identify differentially ASVs for characterizing the differences among the five clusters calculated as reported below in the Statistical Analysis section.

### 5.3. Statistical Analyzes

To categorize the maize silage samples into their quantity and quality of mycotoxin contents, we used a hierarchical cluster analysis using main variables related to mycotoxin contamination (i.e., total count of mycotoxins and concentrations of *Aspergillus*-, *Fusarium*-, *Penicillium*-, *Alternaria*-, and other mycotoxigenic fungi-produced mycotoxins) by the unweighted pair group mean with the arithmetic averages (UPGMA) method by the CLUSTER procedure of SAS (2003). Then, chemical, biological, fermentative measurements, mycotoxins contaminations as well as ASVs at the phylum, order, family, genus, and species levels characterizing corn silages were analyzed as a completely randomized design by using the Generalized Linear Model (GLM) procedure of SAS (2003). The fixed effect of the model was the cluster (*n* = 1 to 5). Mean post-hoc comparisons were performed by using Least-Squares Means (LSMEANS) option of SAS (2003), except for microbiological data for which a Benjamini-Hochberg False Discovery Rate (FDR) was used as a multiple test correction method. To understand the dynamic for individual ASVs, the sequences were manually compared to the National Center for Biotechnology Information (NCBI) database in order to annotate the phylogenic identification. Significance was declared at a *p* < 0.05 and, if biologically retained important, tendency at a 0.05 *< p <* 0.10.

## Figures and Tables

**Table 1:** Counts (*n*) and sums (µg/kg dry matter or DM) of mycotoxins of corn silages belonging to different clusters.

Items	Cluster 1	Cluster 2	Cluster 3	Cluster 4	Cluster 5	√MSE	*p* Value
	*n* = 24	*n* = 22	*n* = 2	*n* = 9	*n* = 7		
**Counts of mycotoxins**	24.7	23.5	42.5	25.4	32.7	5.93	<0.05
**Aspergillus toxins**	3.1	2.6	4.0	2.2	4.1	0.99	<0.05
**Alternaria toxins**	1.0	0.2	2.5	0.3	1.1	1.07	<0.05
**Zearalenone** **and its metabolites**	0.4	0.2	2.0	0.2	0.6	0.55	<0.05
**Trichothecenes type B**	0.8	0.7	1.5	1.0	0.9	0.56	0.256
**Fumonisins and their metabolites**	4.8	5.8	6.5	6.7	7.7	1.46	<0.05
**Enniatins**	0.8	0.3	3.5	0.2	1.0	1.18	<0.05
**Beauvericin**	0.8	1.0	1.0	1.0	1.0	0.24	0.133
**Other Fusarium toxins**	6.5	6.9	11.5	7.2	8.9	1.62	<0.05
**Penicillium toxins**	4.6	4.5	6.5	5.4	6.3	1.10	<0.05
**Other fungi toxins**	0.6	0.1	1.5	0.0	0.9	0.97	0.103
**Unspecified fungi toxins**	0.8	0.1	3.0	0.0	0.7	0.79	<0.05
**Sums of mycotoxins**							
**Aspergillus toxins**	147.0	84.5	565.2	70.3	186.7	104.04	<0.05
**Alternaria toxins**	5.8	4.4	18.7	29.6	18.7	32.67	0.308
**Zearalenone** **and its metabolites**	8.8	4.0	152.8	0.5	41.4	46.27	<0.05
**Trichothecenes type B**	28.8	15.4	192.6	33.5	57.6	41.67	<0.05
**FB and their metabolites**	215.4	339.1	475.3	473.5	1944.9	289.56	<0.05
**Enniatins**	0.6	0.3	3.1	0.5	5.7	4.46	0.075
**Beauvericin**	4.1	8.5	30.8	19.7	27.1	13.15	<0.05
**Other Fusarium toxins**	229.9	755.3	619.7	1564.8	675.1	172.65	<0.05
**Penicillium toxins**	154.6	91.6	708.2	87.3	142.2	107.34	<0.05
**Other fungi toxins**	1.1	0.1	4.3	0.0	4.0	2.85	0.013
**Unspecified fungi toxins**	17.8	1.8	102.0	0.0	26.0	23.51	<0.05

√MSE: root mean square error. When not detectable, the limit of detection of specific mycotoxins was used to compute statistical analysis.

**Table 2:** Concentration ^1^ (µg/kg DM) and incidence (%, within parenthesis) of *Alternaria-* and *Aspergillus*-produced mycotoxin in corn silages belonging to different clusters.

Items	Cluster 1	Cluster 2	Cluster 3	Cluster 4	Cluster 5	√MSE	*p* Value
	*n* = 24	*n* = 22	*n* = 2	*n* = 9	*n* = 7		
**Alternaria mycotoxin count, *n***	**1**	0.2	2.5	0.3	1.1	1.07	0.007
**Altersetin**	6.8 (20.8)	nd (-)	6.9 (50.0)	nd (-)	16.4 (28.6)	7.4	0.358
**Alternariol**	1.9 (20.8)	nd (-)	1.2 (100)	nd (-)	nd (-)	1.41	0.659
**Alternariol, methyl-ether**	1.3 (33.3)	0.6 (4.5)	0.9 (100)	nd (-)	2.9 (14.3)	0.84	0.267
**Tentoxin**	18.2 (4.2)	46.2 (9.1)	nd (-)	88.9 (33.3)	34.3 (14.3)	111.55	0.929
**4Z-Infectopyron**	15.6 (16.7)	3.9 (4.5)	27.5 (50.0)	nd	15.3 (57.1)	12.04	0.617
**Aspergillus mycotoxin count, *n***	3.1	2.6	4	2.2	4.1	0.99	<0.05
**Rugulusovin**	65 (100)	31.1 (100)	310.7 (100)	24.6 (100)	34.7 (100)	53.56	<0.05
**Emodin**	4.9 (50)	1.6 (31.8)	7.2 (100)	1 (11.1)	3.7 (57.1)	2.79	0.066
**Brevianamide F**	74.3 (95.8)	30.6 (95.5)	246.4 (100)	40.5 (88.9)	100.1 (100)	65.65	<0.05
**Kojic acid**	44.1 (16.7)	104 (22.7)	nd (-)	83.5 (11.1)	105.0 (42.9)	86.31	0.734
**3-Nitropropionic acid**	5.2 (12.5)	1.3 (4.5)	nd (-)	2 (11.1)	6.8 (57.1)	6.25	0.824
**Averufin**	0.3 (4.2)	nd (-)	nd (-)	nd (-)	nd (-)	-	-
**Bis(methylthio)gliotoxin**	nd (-)	nd (-)	nd (-)	nd (-)	3 (14.3)	-	-
**Asperphenamate**	nd (-)	0.3 (4.5)	nd (-)	nd (-)	1.7 (14.3)	-	-

nd: mycotoxin not detectable in any samples. √MSE: root mean square error. When not detectable, the limit of detection of specific mycotoxins was used to compute statistical analysis. ^1^ Concentration represented the average values of only detectable mycotoxins.

**Table 3:** Concentration ^1^ (µg/kg DM) and incidence (%, within parenthesis) of regulated and emerging *Fusarium*-produced mycotoxin in corn silages belonging to different clusters.

Items	Cluster 1	Cluster 2	Cluster 3	Cluster 4	Cluster 5	√MSE	*p* Value
	*n* = 24	*n* = 22	*n* = 2	*n* = 9	*n* = 7		
**Fumonisins mycotoxins count, *n***	4.8	5.7	6.5	6.7	7.7	1.46	<0.05
**Fumonisin B1 ^2^**	155 (95.8)	228.7 (100)	181.8 (100)	321.9 (100)	1382.2 (100)	216.24	<0.05
**Fumonisin B2 ^2^**	36.6 (95.8)	67.7 (95.5)	139.1 (100)	96.1 (100)	285 (100)	74.58	<0.05
**Fumonisin B3**	16.3 (75)	19.1 (100)	69 (100)	28.2 (100)	149.6 (100)	39.05	<0.05
**Fumonisin B4**	10.1 (70.8)	17 (95.5)	28.9 (100)	14.9 (100)	68.6 (100)	21.14	<0.05
**Fumonisin B6**	2.9 (8.3)	4.2 (13.6)	nd (-)	2.5 (22.2)	4.2 (71.4)	1.73	0.607
**Fumonisin A1**	4 (54.2)	4.4 (77.3)	9.9 (100)	4.5 (100)	18.1 (100)	6.78	<0.05
**Fumonisin A2**	2.5 (16.7)	4.3 (40.9)	5.6 (50)	2.9 (66.7)	12.4 (100)	3.45	<0.05
**Masked Fumonisin A1**	16.3 (58.3)	8.8 (54.5)	43.8 (100)	7 (77.8)	26 (100)	13.66	<0.05
**Zearalenone metabolites count, *n***	0.4	0.2	2	0.2	0.6	0.55	0.001
**Zearalenone ^2^**	4.1 (29.2)	22 (18.2)	7.1 (100)	2.5 (22.2)	1.7 (42.9)	19.04	0.579
**Zearalenone-sulfone**	91.2 (8.3)	nd (-)	145.7 (100)	nd (-)	284.9 (14.3)	132.85	0.584
**Trichothecenes type-B count, *n***	0.8	0.7	1.5	1	0.9	0.56	0.256
**Deoxynivalenol ^2^**	38.4 (62.5)	22.6 (68.2)	151.7 (100)	33.5 (100)	73.8 (71.4)	41.69	0.002
**Nivalenol**	55 (8.3)	nd (-)	81.9 (50)	nd (-)	34.4 (14.3)	27.73	0.635
**Deoxynivalenol-3-glucoside**	6.7 (4.2)	nd	nd	nd	nd	-	-
**other Fusarium mycotoxins count, *n***	6.5	6.9	11.5	7.2	8.9	1.62	<0.05
**Siccanol**	30 (58.3)	464.4 (100)	210 (100)	1269.2 (100)	255.8 (100)	208.26	<0.05
**Monocerin**	3.9 (16.7)	5.4 (40.9)	19.9 (100)	5.2 (33.3)	3.3 (57.1)	6.726	0.088
**Moniliformin**	10.9 (95.8)	9 (95.5)	39.9 (100)	16.8 (100)	39 (100)	16.53	<0.05
**Equisetin**	8.7 (91.7)	17.7 (90.9)	141.1 (100)	5.4 (100)	8.9 (100)	29.09	<0.05
**Epiequisetin**	8.4 (79.2)	15.1 (90.9)	123.4 (100)	5.1 (100)	9.4 (100)	24.48	<0.05
**Culmorin**	25.7 (42.7)	14.3 (50)	47.5 (100)	14.6 (55.6)	44.2 (71.4)	19.46	<0.05
**15-Hydroxyculmorin**	4.7 (4.2)	nd (-)	nd (-)	nd (-)	nd (-)	-	-
**Butenolide**	12.9 (33.3)	nd (-)	17.8 (50)	nd (-)	17.9 (28.6)	1.92	<0.05
**Bikaverin**	10 (91.7)	18.1 (90.9)	19.4 (100)	15.2 (100)	49.4 (100)	12.75	<0.05
**Apicidin**	nd (-)	nd (-)	4.2 (50)	nd (-)	1.3 (14.3)	-	-
**Antibiotic Y**	11 (12.5)	1.3 (4.5)	15.1 (50)	nd (-)	18.5 (28.6)	15.7	0.833
**7-Hydroxykaurenolide**	2 (8.3)	2.4 (4.5)	7.8 (100)	nd (-)	3.5 (14.3)	4.76	0.681
**Aurofusarin**	8.5 (16.7)	7 (22.7)	5.5 (100)	6.3 (33.3)	6 (71.4)	5.68	0.963
**Fusaric acid**	159.3 (100)	225.0 (100)	195.5 (100)	241 (100)	263.7 (100)	155.94	0.428
**Enniatins-Beauvericin count, *n***	1.7	1.3	4.5	1.2	2	1.21	<0.05
**Enniatin A**	3.2 (8.3)	0.4 (4.5)	nd (-)	nd (-)	nd (-)	4.03	0.676
**Enniatin A1**	0.3 (12.5)	0.5 (4.5)	0.3 (100)	nd (-)	0.6 (14.3)	0.09	0.176
**Enniatin B**	0.6 (33.3)	0.9 (9.1)	0.8 (100)	2.6 (11.1)	3.7 (28.6)	1.44	0.15
**Enniatin B1**	1 (29.2)	2.2 (9.1)	1.5 (100)	1.8 (11.1)	15.2 (28.6)	6.69	0.202
**Enniatin B2**	nd (-)	nd (-)	0.9 (50)	nd (-)	0.6 (28.6)	0.33	0.562
**Beauvericin**	4.9 (83.3)	8.5 (100)	30.8 (100)	19.7 (100)	27.2 (100)	13.57	*p* < 0.05

nd: mycotoxin not detectable in any samples. √MSE: root mean square error. When not detectable, the limit of detection of specific mycotoxins was used to compute statistical analysis. ^1^ Concentration represented the average values of only detectable mycotoxins. ^2^ Regulated mycotoxins by EU legislations.

**Table 4:** Concentration ^1^ (µg/kg of DM) and incidence (%, within parenthesis) of *Penicillium*- and different fungal strains-produced mycotoxins in corn silages belonging to different clusters.

Items	Cluster 1	Cluster 2	Cluster 3	Cluster 4	Cluster 5	√MSE	*p* Value
	*n* = 24	*n* = 22	*n* = 2	*n* = 9	*n* = 7		
**Penicillium mycotoxins count, *n***	4.6	4.5	6.5	5.4	6.3	1.1	0.001
**Skyrin**	nd (-)	nd (-)	nd (-)	nd (-)	6.2 (14.3)	-	-
**Asperglaucide**	nd (-)	nd (-)	nd (-)	1.2 (11.1)	nd (-)	-	-
**Secalonic acid**	4.1 (29.2)	5.3 (50)	nd (-)	7.3 (33.3)	17.9 (57.1)	10.78	0.214
**7-Hydroxypestalonic**	1.2 (4.2)	1.4 (22.7)	nd (-)	1.7 (44.4)	2.6 (28.6)	0.42	0.045
**Chlorocitreorosein**	nd (-)	nd (-)	1.3 (50)	nd (-)	nd (-)	-	-
**Pestalotin**	3.4 (45.8)	3.9 (27.3)	26.5 (100)	3 (100)	5.2 (85.7)	4.29	-
**Oxaline**	5.7 (62.5)	8 (63.6)	3.8 (100)	4.8 (55.6)	9.9 (85.7)	5.23	0.314
**Flavoglaucin**	10.4 (8.3)	2.5 (9.1)	nd (-)	nd (-)	nd (-)	9.46	0.488
**Cyclopenin**	1 (25)	nd (-)	1 (100)	nd (-)	nd (-)	0.58	0.972
**Fellutanine A**	109.1 (100)	54 (100)	577.6 (100)	49.9 (100)	55.5 (100)	79.6	-
**Mycophenolic acid**	5.7 (4.2)	nd (-)	nd (-)	nd (-)	43.5 (28.6)	21.52	0.388
**Mycophenolic acid IV**	0.5 (4.2)	nd (-)	nd (-)	nd (-)	2.3 (28.6)	2.09	0.6
**Phenopyrrozin**	23.3 (100)	18.1 (95.5)	47.1 (100)	10 (100)	15.6 (100)	24.67	0.339
**Questiomycin A**	18.3 (79.2)	12.7 (86.4)	51.7 (100)	18.4 (100)	33.2 (100)	17.67	0.015
**Metabolites from different fungal strains count, *n***	0.6	0.1	1.5	-	0.9	0.97	0.103
**Ilicicolin A**	0.8 (4.2)	0.3 (4.5)	nd (-)	nd (-)	nd (-)	-	-
**Ilicicolin B**	1.1 (4.2)	nd (-)	nd (-)	nd (-)	1.7 (14.3)	-	-
**Citreorosein**	3.4 (12.5)	nd (-)	3.9 (50)	nd (-)	4.6 (28.6)	1.74	0.775
**Ascochlorin**	1.3 (20.8)	1.4 (4.5)	3.9 (50)	nd (-)	7.7 (14.3)	0.88	0.011
**Bassianolide**	5.2 (4.2)	nd (-)	nd (-)	nd (-)	6.3 (14.3)	-	-
**Ascofuranone**	0.5 (12.5)	0.4 (4.5)	0.8 (50)	nd (-)	3.2 (14.3)	0.24	0.028
**Macrosporin**	1.5 (12.5)	nd (-)	nd (-)	nd (-)	nd (-)	-	-
**Unspecific Metabolites count, *n***	0.8	0.1	3	nd	0.7	0.79	<0.05
**Iso-rhodoptilometrin**	0.8 (33.3)	1.1 (4.5)	1.8 (100)	nd (-)	0.8 (28.6)	0.63	0.294
**Norlichexanthone**	1 (8.3)	nd (-)	2 (100)	nd (-)	5.4 (14.3)	0.64	0.059

nd: mycotoxin not detectable in any samples. √MSE: root mean square error. When not detectable, the limit of detection of specific mycotoxins was used to compute statistical analysis. ^1^ Concentration represented the average values of only detectable mycotoxins.

**Table 5:** Chemical, biological and fermentative traits, fermentative quality scores, and silage making procedures characterizing corn silages belonging to different clusters.

Items	Cluster 1	Cluster 2	Cluster 3	Cluster 4	Cluster 5	√MSE	*p* Value
	*n* = 24	*n* = 22	*n* = 2	*n* = 9	*n* = 7		
**Chemical parameters (% DM)**							
**DM (% fresh matter)**	34.7	34.3	37.9	33.5	31.3	4.56	0.342
**Ash**	5.7	5.7	5.7	6.0	5.8	0.53	0.553
**CP**	8.1	8.2	7.7	8.4	8.9	0.87	0.230
**EE**	2.9	3.0	3.0	2.8	3.0	0.21	0.351
**NDF**	37.7	37.1	35.5	39.0	36.9	2.40	0.180
**ADF**	25.2	24.7	23.3	25.8	24.7	1.86	0.389
**ADL**	2.8	2.9	3.2	3.1	3.0	0.32	0.281
**NDIP**	1.1	1.1	0.8	1.1	1.0	0.25	0.507
**ADIP**	0.8	0.8	0.6	0.7	0.8	0.16	0.441
**24 h NDFD (% NDF)**	52.1	50.9	47.6	51.9	50.7	3.19	0.303
**Starch**	31.2	31.5	36.1	28.7	30.2	3.44	0.067
**Fermentative parameters (% DM)**							
**pH (dmnl)**	3.67	3.76	3.84	4.02	3.83	0.331	0.156
**Acetic acid**	3.42	2.87	3.28	2.54	3.82	1.30	0.223
**Propionic acid**	0.14	0.13	0.04	0.17	0.41	0.19	<0.05
**Butyric acid**	0.008	0.003	0.001	0.005	0.006	0.011	0.750
**Lactic acid**	3.29	4.02	1.91	3.06	3.78	1.612	0.246
**Lactic to Acetic**	1.2	2.2	0.6	1.5	1.1	1.70	0.273
**Ethanol**	0.6	0.5	0.6	0.6	0.3	0.48	0.779
**Lactic to (Acetic/Ethanol)**	0.91	1.78	0.50	1.30	0.99	1.57	0.376
**1,2 propanediol**	0.60	0.49	0.73	0.50	0.17	0.289	<0.05
**N-NH3 (% TN)**	11.38	11.71	7.78	7.85	13.58	7.737	0.569

dmnl = dimensionless; CP = crude protein; EE = ether extract; NDF = neutral detergent fiber; ADF = acid detergent fiber; ADL = lignin; NDIP = neutral detergent insoluble protein; ADIP = acid detergent insoluble protein; 24 h NDFD = NDF disappearance evaluated in situ after 24 h of rumen incubation; N-NH3 = ammonia nitrogen; TN = total nitrogen. √MSE: root mean square error.

## Supplementary Materials

The following are available online at https://www.mdpi.com/2072-6651/13/3/232/s1, Figure S1: relative abundance of Amplicon Sequence Variance (ASVs) as the genomic features characterizing the differences between clusters for specific bacteria.
