# Supplementary material for: Co-Occurrence of Regulated and Emerging Mycotoxins in Corn Silage: Relationships with Fermentation Quality and Bacterial Communities
Source: Toxins (Basel). 2021 Mar 23;13(3):232. doi: 10.3390/toxins13030232 (PMC8004697; doi:10.3390/toxins13030232)
Supplement: Supplementary file 1 [file toxins-13-00232-s001.pdf]

Supplementary Materials: Co-Occurrence of Regulated and Emerging Mycotoxins in Corn Silage: Relationships with Fermentation Quality and Bacterial Communities

Antonio Gallo, Francesca Ghilardelli, Alberto Stanislao Atzori, Severino Zara, Barbara Novak, Johannes Faas and Francesco Fancello

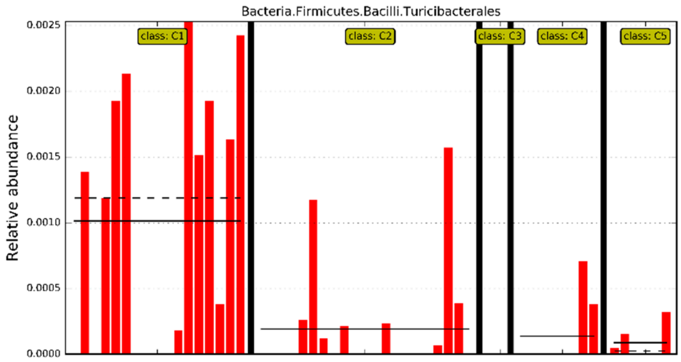

(**a**)

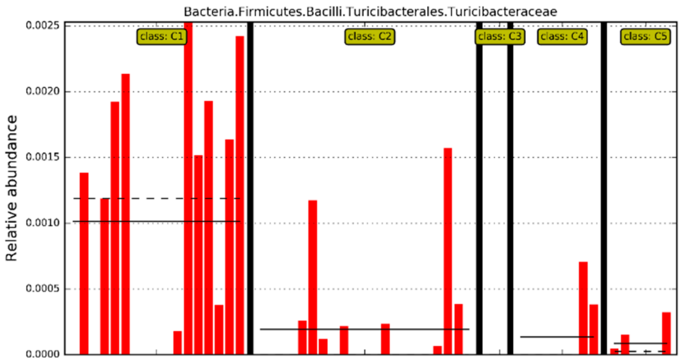

(**b**)

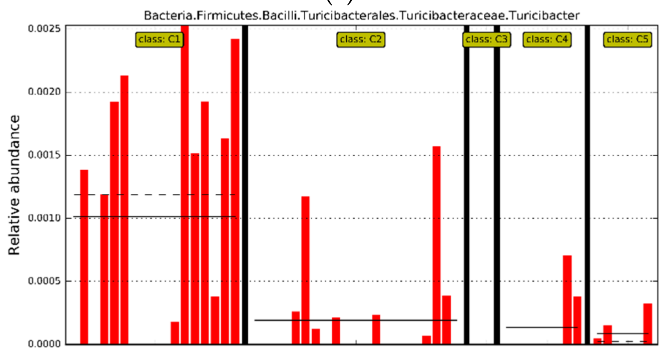

(**c**)

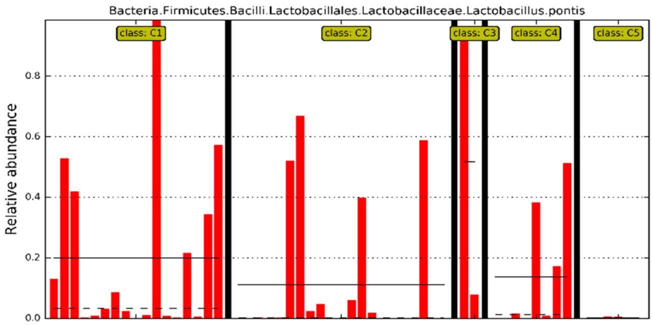

(**d**)

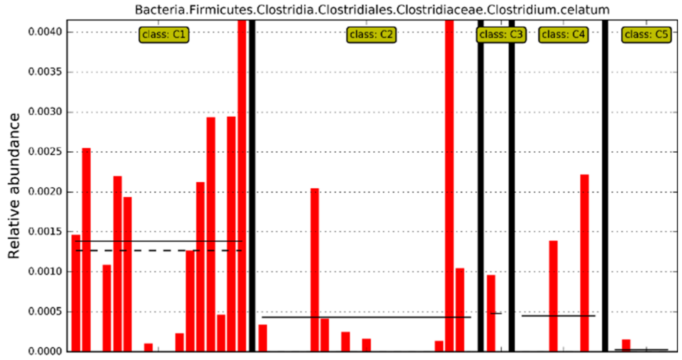

(**e**)

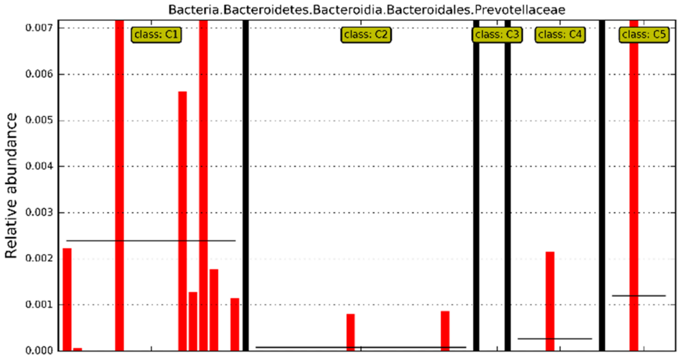

(**f**)

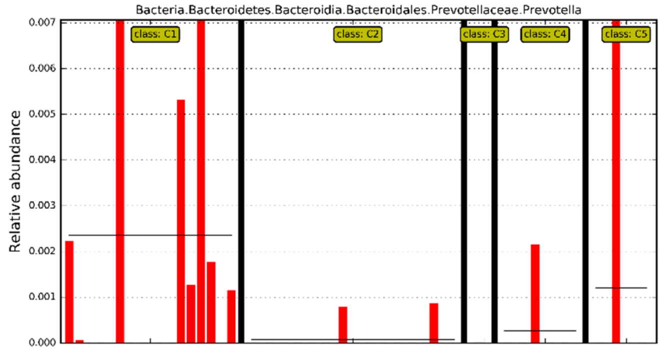

(g)

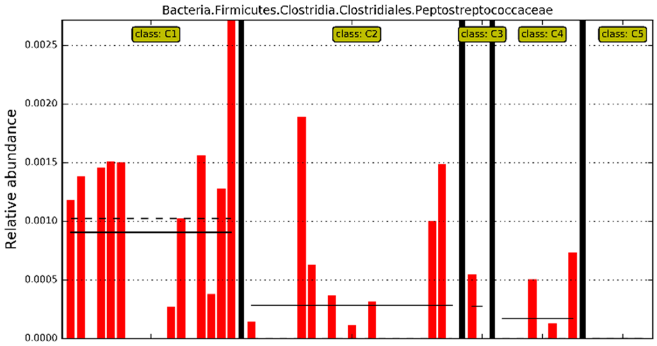

(h)

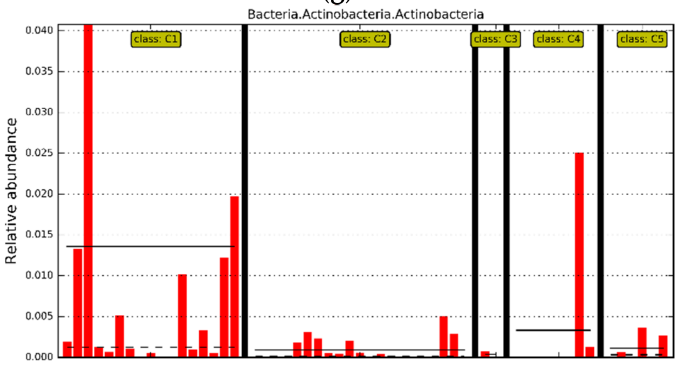

(i)

Figure S1. Linear discriminant analysis (LDA) combined with effect size measurements (LEfSe) revealed 9 Amplicon Sequence Variances (ASVs) that allow discrimination among the five clusters found (based to content and type of mycotoxins). A *p*-value of <0.05 and a score ≥2.0 were considered significant in Kruskal–Wallis and pairwise Wilcoxon tests, respectively. The horizontal straight line in the panel indicates the cluster means, and the dotted line indicates the cluster medians.
